# Targeting SHP2 phosphatase in breast cancer overcomes RTK-mediated resistance to PI3K inhibitors

**DOI:** 10.1186/s13058-022-01521-3

**Published:** 2022-04-01

**Authors:** Guus J. J. E. Heynen, Kamil Lisek, Regina Vogel, Annika Wulf-Goldenberg, Joshua Alcaniz, Elodie Montaudon, Elisabetta Marangoni, Walter Birchmeier

**Affiliations:** 1 Max Delbrück Center for Molecular Medicine (MDC) in the Helmholtz Society, Campus Berlin-Buch, Robert-Rössle-Str. 10, 13125 Berlin, Germany; 2 Experimental and Pharmacological Oncology (EPO), Campus Berlin-Buch, Building 82, Robert-Rössle-Str. 10, 13125 Berlin, Germany; 3 Preclinical Investigation Laboratory, Institut Curie, 20 Rue d’Ulm, 75248 Paris, France

**Keywords:** Breast cancer, Drug resistance, Targeted therapy, PI3K and MAPK signaling, SHP2

## Abstract

**Background:**

PI3K signaling is frequently activated in breast cancer and is targeted by PI3K inhibitors. However, resistance of tumor cells to PI3K inhibition, often mediated by activated receptor tyrosine kinases, is commonly observed and reduces the potency of PI3K inhibitors. Therefore, new treatment strategies to overcome resistance to PI3K inhibitors are urgently needed to boost their efficacy. The phosphatase SHP2, which plays a crucial role in mediating signal transduction between receptor tyrosine kinases and both the PI3K and MAPK pathways, is a potential target for combination treatment.

**Methods:**

We tested combinations of PI3K and SHP2 inhibitors in several experimental breast cancer models that are resistant to PI3K inhibition. Using cell culturing, biochemical and genetic approaches, we evaluated tumor cell proliferation and signaling output in cells treated with PI3K and SHP2 inhibitors.

**Results:**

Combination treatment with PI3K and SHP2 inhibitors counteracted both acquired and intrinsic breast cancer cell resistance to PI3K inhibition that is mediated by activated receptor tyrosine kinases. Dual PI3K and SHP2 inhibition blocked proliferation and led to sustained inactivation of PI3K and MAPK signaling, where resistant cells rapidly re-activated these pathways upon PI3K inhibitor monotreatment. In addition, we demonstrate that overexpression of SHP2 induced resistance to PI3K inhibition, and that SHP2 was frequently activated during the development of PI3K inhibitor resistance after prolonged treatment of sensitive cells.

**Conclusions:**

Our results highlight the importance of SHP2 as a player in resistance to PI3K inhibitors. Combination treatment with PI3K and SHP2 inhibitors could pave the way for significant improvements in therapies for breast cancer.

**Supplementary Information:**

The online version contains supplementary material available at 10.1186/s13058-022-01521-3.

## Background

Breast cancer is the most prevalent form of cancer in women and one of the leading causes of morbidity for women in the Western world, accounting for up to 15% of cancer deaths [1]. Gene expression and whole genome sequencing have exposed five main subtypes of breast tumors: luminal A and B, HER2±, normal-like, and triple negative (TNBCs, or basal-like) breast cancers [2, 3]. Luminal breast cancers express the estrogen and/or progesterone hormone receptor, while HER2 + tumors overexpress HER2, making these subtypes vulnerable to targeted (hormonal) therapies. In contrast, TNBC’s do not express hormone receptors, which severely hampers treatment options [4]. Frequent gene alterations in breast cancer are found in the *TP53* and *PIK3CA* genes; other mutations activate receptor tyrosine kinase (RTK) signaling [2]. A high proportion of breast cancers also exhibits high activation of Wnt/β-catenin signaling [5, 6].

Recently, new classes of cancer drugs have been developed that target proteins or pathways, which have been rendered constitutively active by mutations and promote cancer cell proliferation and survival. Most of these compounds target components of the MAPK, PI3K and Wnt pathways [5, 7]. While initially, they may elicit positive responses and significantly slow tumor progression, often the effects are not sustained [8, 9]. In certain cases, tumor cells appear to be intrinsically resistant, while in others, over the long term, they acquire resistance to the drugs [10]. This has created a pressing need for new treatment options [11].

Recent work has shown that tumor cells often evade therapies by finding alternative ways to re-activate the pathways that have been targeted. They achieve this through elaborate feedback mechanisms, autocrine and paracrine signaling, and other means [12]. In many cases this involves the activation of one or multiple RTKs. The EGF receptor for example, is activated via a negative feedback loop in BRAF mutant colon cancer upon treatment with the BRAF inhibitor vemurafenib [13, 14]. MET receptor activation by its ligand HGF leads to resistance to a range of targeted therapies [15–17]. These resistance mechanisms warrant the development of combination therapies.

Our search for potential targets has led to the Src homology region 2 domain-containing phosphatase 2 (SHP2), encoded by the *PTPN11* gene. SHP2 is crucial in development and is an important mediator of MAPK and PI3K signaling downstream of the ErbB and FGF receptors and other RTKs [18–20]. SHP2 has been implicated in breast carcinogenesis [21], and SHP2 mutations are found in several types of solid tumors as well as in two syndromes, Leopard and Noonan syndrome, where it makes patients prone to multiple types of cancer [19, 22, 23]. In addition, RTK-mediated resistance to cancer drugs that target the MAPK pathway is dependent on SHP2 [24–26]. This makes SHP2 a highly attractive drug target and has prompted the development of a number of SHP2-specific inhibitors [27–30]. Several preclinical studies have shown promise for their use as cancer treatments. In tumor cells, activated RTKs mediate resistance to BRAF, MEK and ALK inhibitors; combining these with SHP2 inhibitors is a powerful strategy to overcome resistance to these drugs [24, 25, 31].

Breast cancers often exhibit a deregulation of the PI3K/AKT/mTOR pathway [32]. PI3K signaling can be aberrantly activated by, for instance, overexpression of the HER2 receptor tyrosine kinase [33]. Activating mutations in *PIK3CA,* the gene that encodes the catalytic subunit of the PI3K enzyme (most notably residues 545 and 1047), are frequently found in luminal and HER2 + breast cancer cells. Deletion of the phosphatase and tensin homolog (*PTEN*) tumor suppressor gene, a negative regulator of PI3K signaling, is  also recurrently detected in breast cancer cells [2]. PI3K/AKT/mTOR activation contributes to carcinogenesis by increasing cell proliferation and enhancing cell survival, metastasis and metabolic activity. This has led to significant efforts to develop PI3K inhibitors that target the PI3K enzyme and subsequent downstream signaling, and a number of these are currently in preclinical and clinical development [34]. One prominent example of a clinically approved PI3K inhibitor is BYL719 (alpelisib), which is given in combination with fulvestrant to patients with hormone receptor-positive, HER2-negative advanced breast cancer harboring *PIK3CA* mutations [35] and is being evaluated for other indications in ongoing clinical trials. PI3K mutant breast cancers often initially respond to PI3K inhibitor therapy. However, tumors readily develop resistance or are intrinsically resistant to PI3K inhibitors [36]. Strategies to overcome this are urgently needed. Many of the mechanisms underlying resistance to PI3K inhibitors are mediated by growth factors and activated RTKs [37–40]. In the present study, we demonstrate that combination treatment with BYL719 and SHP2 inhibitors overcomes RTK-mediated resistance to BYL719 in both luminal and triple-negative breast cancer cells, which is a crucial finding to improve the efficacy of PI3K inhibitors.

## Methods

### Cell lines and cell culturing

T47D, MDA-MB-453, MDA-MB-486, BT549 and CAL-51 cells were purchased from the Deutsche Sammlung von Mikroorganismen und Zellkulturen (DSMZ). MCF7 cells were purchased from ATCC. HCC1806 and HCC1937 cells were kind gifts of Rene Bernards, Amsterdam. SUM159 cells were a kind gift of Volker Haucke, Berlin. MCF7 and T47D cells were cultured in DMEM (Life Technologies) supplemented with 10% FBS, 5 μg/mL insulin (Sigma, cat.# I0516) and 1% penicillin/streptomycin (Sigma). MDA-MB-453, MDA-MB-468, BT549 and CAL-51 cells were cultured in DMEM supplemented with 10% FBS, 1% non-essential amino acids (Gibco, 11140050) and 1% penicillin/streptomycin. HCC1806 and HCC1937 cells were cultured in RPMI-1640 (Life Technologies) supplemented with 10% FBS and 1% penicillin/streptomycin. SUM159 cells were cultured in Ham/F12 (Life Technologies), 10% FCS, 1 μg/ml hydrocortisone, 10 mM HEPES (Gibco, 11560496), 5 μg/ml insulin and 1% penicillin/streptomycin. All cell lines were cultured at 37 °C and 5% CO_2_ in a humidified incubator. See Additional file 1: Table S1 for a summary on cell line characteristics [41, 42].

### Colony formation assays

Cells were seeded at 5000 cells/well in 12-well plates (Sarstedt) and allowed to adhere overnight. For long-term colony formation assays with MCF7, T47D and MDA-MB-453 cells, 50,000 cells were seeded in a 10 cm dish and allowed to adhere overnight. The next day, compounds were added as indicated and were refreshed every 3–4 days. When vehicle (DMSO)-treated cells (indicated as ‘UT, untreated’) reached confluence, wells were fixed in 3.7% formaldehyde, stained with 0.1% crystal violet and subsequently scanned. To quantify crystal violet staining, 10% acetic acid was used to dissolve crystal violet for 15 min on a shaker. The resulting acidic acid/crystal violet solution was then diluted 1:4 in water and absorbance at 590 nm was measured by photospectometer. Experiments were performed three times, and representative results are shown.

### 3D organoid formation assay

HBCx4B and HBCx60 patient-derived xenografts (PDXs) were established from two *PIK3CA*-mutated triple-negative breast cancers by grafting tumor fragments into the interscapular fat pad of nude mice as previously described [43]. Once the tumors had formed, they were harvested, and viable epithelial cells were isolated. HBCx4B and HBCx60 cells were seeded in 25 μl droplets containing 50% reduced growth factor-Matrigel at a density of 100cells/μl. Plates were carefully flipped and Matrigel was let to solidify at 37 °C for 30–45 min. 0.5 ml of stem medium MEBM (Lonza Cat. #CC-3151 supplemented with 2% B27 (Invitrogen, Cat. # 17504044), 20 ng/ml bFGF (Invitrogen, Cat. #13256029), 20 ng/ml EGF (Sigma, Cat. #SRP3196-500 μg), 4 μg/ml heparin (Sigma, Cat. #H3149), 5 µg/ml insulin (Sigma, Cat. #I0516-5 ml), 0.5 µg/ml hydrocortisone (Sigma, Cat. #H0888-1G) and 1X Gentamicin (Sigma, Cat. #G1397-100ML) was added per well of 24-well plates containing a 25 μl droplet with cells. Medium was changed every second day with treatments as indicated. After 10 days of growth, microscopic images were acquired with a bright-field Leica microscope. Quantification of organoids was performed by analyzing overview pictures with automatic colony count with ImageJ software.

### CellTiter-Glo cell viability assay

3D cultures were seeded into 96-well opaque-walled plates. Control and treated spheres with 100 μl of medium per well were incubated for 30 min at room temperature. 100 μl of CellTiter-Glo reagent was added to each well and incubated for 2 min shaking to induce cell lysis. Plates were then rested for 10 min at room temperature to stabilize the luminescent signal. Luminescence was subsequently measured on a luminometer.

### Plasmids, cloning and transfection

To generate CRISPR/Cas9 *PTPN11* constructs, the pX458 vector was used to clone in two independent gRNAs that target the *PTPN11* gene. The primer sequences for both *PTPN11* gRNAs are as follows: *PTPN11* gRNA 1: Fw: CACCGGAGGAACATGACATCGCGG, Rev: AAACCCGCGATGTCATGTTCCTCC; *PTPN11* gRNA 2: Fw: CCACGAACATGACATCGCGGAGGTG, Rev: AAACCACCTCCGCGATGTCATGTTC. Forward and reverse oligos for each gRNA were annealed and ligated into Bbs1-digested pX458 vector. Target cells were subsequently transfected with the pX458-PTPN11-gRNA plasmids using polyethylenimine (PEI). Positively transfected cells expressing GFP were then FAC-sorted as single cells in 96-well plates und cultured in gentamycin-containing medium to prevent FAC-sorting-related contamination. Clones were allowed to grow and analyzed for the SHP2 status. SHP2 knockout clones were named after gRNA and clone name, e.g., T47D 1.D = gRNA1, clone D.

SHP2 overexpression and reconstitution experiments: the pCMV-EGFP plasmid was available in the Birchmeier lab, pCMV-SHP2-WT (#8381) and pCMV-SHP2-C459S (#8382) plasmids were purchased from AddGene.

MCF7 wild type cells and HCC1806 SHP2 knockout clone 1.C were transfected with pCMV-GFP, pCMV-SHP2-WT or pCMV-SHP2-C459S using polyethylenimine (PEI). Subsequently, transfected cells were selected with G418 (800 µg/ml G418 until non-transfected control cells were dead, then maintained in 200 µg/ml G418) and clones that formed were picked individually, subcultured and analyzed for SHP2 expression. Clones with SHP2 expression levels closest to SHP2 expression in parental cells were selected for further experimenting.

### BYL719-resistant T47D and MDA-MB-453 cells

T47D and MDA-MB-453 cells were seeded at low density in 10 cm dishes (100,000 cells/dish) and treated with BYL719 (2 and 1 μM, respectively) until resistant colonies formed. BYL719-resistant clones were picked and subcultured individually in BYL719-containing medium for subsequent analysis.

### Reagents, inhibitors, growth factors and antibodies

Reagents were: Insulin (Sigma, I0516), NEAA (Gibco, 11140050), gentamycin (Sigma, G1272), G418 (OZ Biosciences, GS21000).

Inhibitors were: BYL719 (Selleckchem, S2814), SHP099 (Selleckchem, S8278), GDC-0941 (Selleckchem, S1065), GS-493 (Millipore, 538099). All inhibitors were solved in DMSO.

Growth factors were: NRG1 (396-HB-050), EGF (236-EG-200) and FGF10 (345-FG-025), purchased from R&D Systems.

Antibodies: AKT (Cell Signaling, 9272), p-AKT S473 (Cell Signaling, 4060), p-4E-BP1 T37/46 (Cell Signaling, 2855), ERK (Cell Signaling, 9102), p-HER3 Y1289 (Cell Signaling, 4791), p-MET Y1234/1235 (Cell Signaling, 3077), p-FGFR Y653/654 (Cell Signaling 3471), p-S6 ribosomal protein S240/244 (Cell Signaling, 5364), p-ERK Y204 (Santa Cruz, sc-7383), HSP90 (Santa Cruz, sc-13119), SHP2 (Santa Cruz, sc-280), p-SHP2 Y542 antibody (AbCam, 62322), p-EGFR Y1068 (AbCam, 5644). Secondary rabbit (#111-035-144) and mouse (#115-035-062) antibodies were from Dianova.

### Western blotting and human Phospho-RTK array

Cell samples were lysed in RIPA buffer (50 mM Tris pH7.4, 150 mM NaCl, 1% NP40, 0.1% SDS and 0.5% Sodium deoxycholate) supplemented with protease inhibitor (Roche, #11836153001) and phosphatase inhibitor cocktails 2 and 3 (Sigma-Aldrich, #P57261 and #P0044). Protein concentrations were determined by Pierce BCA protein assay kit (Thermo Scientific, #23225). Proteins were separated by SDS-PAGE in Laemmli buffer (0.25 M Tris, 1.92 M glycine, 1% SDS), transferred to PVDF membranes (Carl Roth, pore size 0.45 μM) in transfer buffer (25 mM Tris, 192 mM glycine, 20% methanol) and subsequently incubated overnight at 4 °C with indicated antibodies in 5% BSA in PBST. ECL (Perkin Elmer, NEL104001EA) was used to detect antibodies in a Vilber Fusion FX. Western blotting experiments were performed three times and representative results are shown.

The human Phospho-RTK Array was purchased from R&D Systems (ARY001B) and was used according to the manufacturer’s protocols. Cells were seeded in 10 cm dishes, allowed to adhere overnight and subsequently treated as indicated.

### In vivo xenograft experiment

2 × 10^6^ HCC1806 cells were suspended in 20μL PBS and 20μL Matrigel and were injected in the mammary fat pad of 12 female NMRI nude mice. The grafted cells were allowed to grow and form tumors for 10 days, when tumor sizes were ~ 200 mm^3^. At day 10, mice were randomized into 4 groups, Groups A-D, and treatment was started. Group A: vehicle (0.6% methylcellulose, 0.5% Tween80 in 0.9% saline); Group B: BYL719 (15 mg/kg); Group C: SHP099 (75 mg/kg); Group D: BYL plus SHP099 (15 and 75 mg/kg). Treatments were given orally and daily. BYL719 was solved in 0.5% methylcellulose and SHP099 in 0.6% methylcellulose, 0.5% Tween80 in 0.9% saline, which also served as the vehicle treatment. Tumor volumes were measured by calliper on days 10, 13, 17, 20 and 24 after engraftment of the cells. Body weights were measured on the same days. Due to weight loss, the body weight of group D was additionally measured on days 19 and 21 for closer monitoring. After 24 days, tumor burden in the vehicle-treated mice necessitated termination of the experiment and mice were killed.

### Quantifications and statistical analysis

Colony formation experiments were quantified by measuring crystal violet intensity of each individual well. Results of three independent colony formation experiments were averaged. Significant differences between indicated conditions were calculated by one-way ANOVA using GraphPad Prism 8 and are indicated with *(*p* ≤ 0.05), **(*p* ≤ 0.01), ***p ≤ 0.001) or ****p ≤ 0.0001).

Image J was used to quantify band intensity of selected western blots and intensities were normalized to band intensity of HSP90 loading control. Results of three western blot experiments were averaged. Significant differences (*p* ≤ 0.05) between untreated and treated conditions were calculated by one-way ANOVA using GraphPad Prism 8 and are indicated with an *.

The statistical analysis used for the organoid and xenograft experiments are described in the figure legends, along with the number of samples analyzed and the plotted error types. Statistical analysis was performed and *p*-value thresholds were obtained using GraphPad 8. No statistical method was used to predetermine sample sizes. The mice in the xenograft experiment were randomized before treatment.

## Results

### Inhibiting SHP2 counteracts acquired resistance to PI3K inhibition in PI3K mutant breast cancer cells

**Fig. 1:**
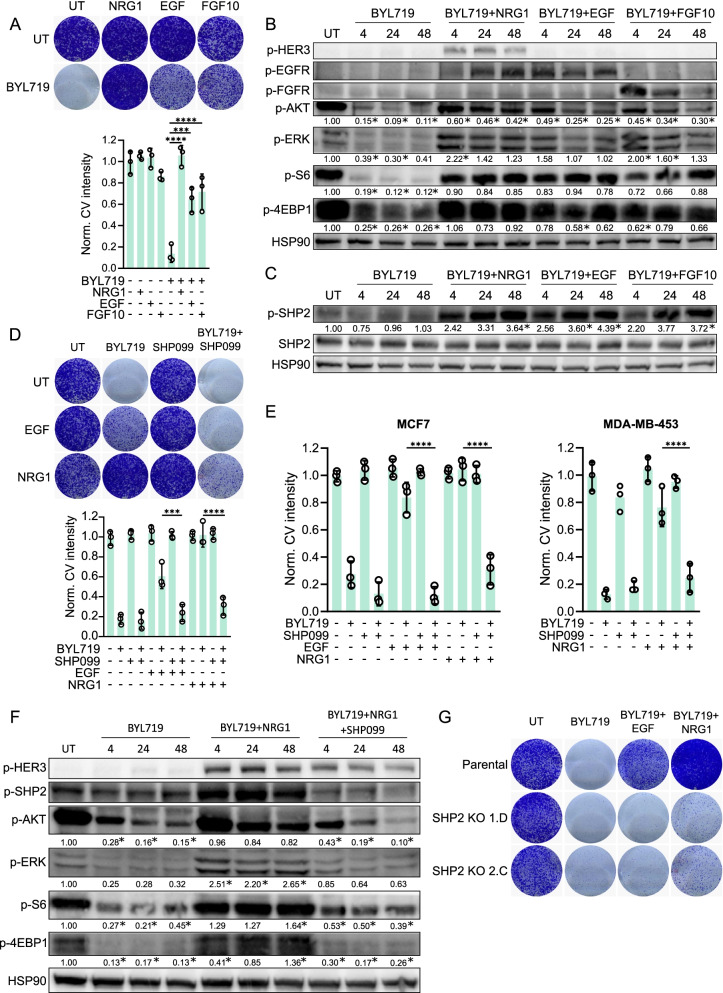
Inhibition of SHP2 counteracts acquired resistance to PI3K inhibition in PI3K mutant breast cancer cells. **a** Multiple growth factors confer resistance to BYL719 in PI3K mutant T47D breast cancer cells. Top: colony formation. Bottom: quantification of crystal violet staining intensity of three independent colony formation experiments. Significance between indicated conditions was calculated by one-way ANOVA. ***p ≤ 0.001, ****p ≤ 0.0001. pBYL719: 2 μM. NRG1, EGF and FGF10: 50 ng/ml. UT: vehicle-treated. **b** Western blot showing the biochemical effects on PI3K and MAPK signaling after BYL719, NRG1, EGF or FGF10 treatments in T47D cells for 4, 24 or 48 h. BYL719: 2 μM. NRG1, EGF and FGF10: 50 ng/ml. Relative gel band intensities of phosphorylated proteins are indicated below each row. Significant differences (*p* ≤ 0.05) in signaling in comparison to the untreated condition are marked with an *. **c** Western blot showing SHP2 phosphorylation of Y542 upon NRG1, EGF and FGF10 treatment in T47D cells. BYL719: 2 μM. NRG1, EGF and FGF10: 50 ng/ml. Significant differences (*p* ≤ 0.05) in induction of SHP2 phosphorylation in comparison to the untreated condition are marked with an *. **d** SHP099 reverses growth factor-induced BYL719 resistance in T47D. Top: colony formation. Bottom: quantification of crystal violet staining intensity of three colony formation experiments. Significance between indicated conditions was calculated by one-way ANOVA. ***p ≤ 0.001, ****p ≤ 0.0001. BYL719: 2 μM. EGF and NRG1: 50 ng/ml. SHP099: 5 μM. **e** SHP099 reverses growth factor-induced BYL719 resistance in MCF7 and MDA-MB-453 cells. Quantifications of crystal violet staining intensity of three colony formation experiments are shown. Significance between indicated conditions was calculated by one-way ANOVA. ****p ≤ 0.0001. BYL719: 5 μM (MCF7) and 1 μM (MDA-MB-453). EGF and NRG1: 50 ng/ml. SHP099: 5 μM. **f** Western blot showing the biochemical effects on PI3K and MAPK signaling after BYL719, NRG1 and SHP099 treatment in T47D cells. BYL719: 2 μM. NRG1: 50 ng/ml. SHP099: 5 μM. Significant differences (*p* ≤ 0.05) in signaling in comparison to the untreated condition are marked with an *. **g** Colony formations with two independent T47D SHP2 knockout clones treated with BYL719 (2 μM), NRG1 and EGF (both 50 ng/ml)

Tumor cells frequently acquire resistance to cancer therapies, including those that target MAPK and PI3K signaling, by activating RTKs by growth factor stimulation [15–17]. We examined the capacity of several growth factors to convey RTK-mediated acquired resistance to BYL719 (alpelisib), a PIK3CA-specific inhibitor that has recently entered the clinic [44], in two luminal breast cancer cell lines with *PIK3CA* mutations, MCF7 and T47D, and in MDA-MB-453, a PI3K mutant HER2 + breast cancer cell line (Table S1). These cell lines were sensitive to BYL719 treatment. However, this sensitivity markedly decreased upon the addition of neuregulin 1 (NRG1), epidermal growth factor (EGF) or fibroblast growth factor 10 (FGF10) (Fig. 1A, Additional file 1: Fig. S1A).

Western blots showed that inhibition of PI3K by BYL719 led to a marked decrease in phosphorylation of the downstream effectors AKT, S6 ribosomal protein and 4E-BP1. In contrast, cells treated with the combination of BYL719 and NRG1, EGF or FGF10 exhibited sustained AKT, S6 ribosomal protein and 4E-BP1 phosphorylation, indicating that PI3K signaling was re-activated. We also detected activation of MAPK signaling through an enhanced phosphorylation of ERK in these cells (Fig. 1B, Additional file 1: Fig. S1B). Phosphorylation of HER3, EGFR and FGFR after treatment with, respectively, NRG1, EGF and FGF10, confirmed RTK activation. Thus, several growth factors have the capacity to induce resistance to BYL719 and re-activate PI3K and MAPK signaling after monotreatment with the inhibitor.

Previous studies have shown that inhibition of the phosphatase SHP2 overcomes resistance to BRAF and MEK mediated by receptor tyrosine kinases [24–26]. We reasoned that in breast cancer cells, the same approach might overcome resistance to PI3K inhibition induced by growth factors. Indeed, we observed an increase in SHP2 phosphorylation and thus activation at the activating tyrosine residue 542 [45] (marked by p-SHP2) in cells treated with BYL719 and NRG1, EGF or FGF10 (Fig. 1C, Additional file 1: Fig. S1B). We applied an allosteric SHP2 inhibitor [29], SHP099, to the three cell lines. This reversed growth factor-driven resistance to BYL719 and blocked cell proliferation in the cell lines (Fig. 1D, E). We used western blotting to analyze the biochemical effects of BYL719, SHP099 and NRG1 combination treatment. This showed significantly lower levels of re-activation of PI3K and MAPK signaling compared to treatment with solely BYL719 and NRG1. The effects of SHP099/NRG1/BYL719 treatment were similar to BYL719 monotreatment, as observed by the low levels of phosphorylation of AKT, S6 ribosomal protein, 4E-BP1 and ERK (Fig. 1F, Additional file 1: Fig. S1C). In addition, diminished SHP2 phosphorylation upon application of SHP099 indicated inhibition of SHP2. Consistently, combining SHP099 with GDC0941 (pictilisib), another PI3K inhibitor [46], or BYL719 with the catalytic inhibitor GS493 (another SHP2 inhibitor) [27], also reversed the resistance to PI3K inhibition driven by growth factors (Additional file 1: Fig. S1D, E). To genetically confirm the results with SHP099 treatment, we used the CRISPR-Cas9 technology to knock out *PTPN11*, the gene encoding SHP2, in T47D cells [47]. Western blotting confirmed the SHP2 knockout status (Additional file 1: Fig. S1F). Treating SHP2 knockout cells with BYL719, NRG1 or EGF reversed the resistant phenotype seen in parental cells (Fig. 1G).

It has been previously shown that tumor cells may acquire resistance to targeted drugs by simultaneously activating several RTKs [48, 49]. This is a major clinical problem, because treatments that combine multiple RTK inhibitors may enhance their toxicity. Since SHP2 controls signal transduction downstream of many RTKs, we wondered whether the inhibition of SHP2 alone might prevent drug resistance mediated by the simultaneous activation of multiple RTKs [18, 19]. To mimic this, we treated MCF7 cells simultaneously with NRG1, EGF and FGF10, which led to strong BYL719 resistance. However, the addition of SHP099 reversed the resistant phenotype (Additional file 1: Fig. S1G). These results demonstrate that using SHP099 to inhibit SHP2 prevents growth factor-driven resistance to PI3K inhibitors in *PIK3CA* mutant breast cancer cells.

### SHP2 confers resistance to PI3K inhibition upon overexpression and long-term PI3K inhibitor exposure

**Fig. 2:**
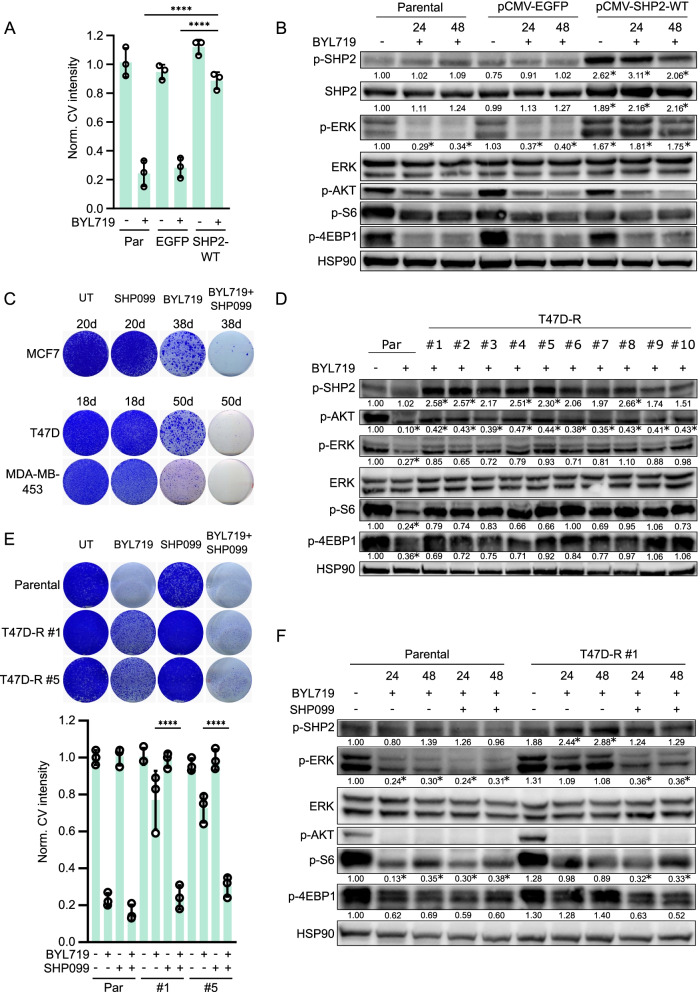
SHP2 confers resistance to PI3K inhibition upon overexpression and long-term PI3K inhibitor exposure. **a** MCF7 cells overexpressing wild type SHP2 (SHP2-WT) are resistant to BYL719 (5 μM) treatment. MCF7-pCMV-EGFP (EGFP) cells serve as control. Quantification of crystal violet staining intensity of three colony formation experiments is shown. Significance between indicated conditions was calculated by one-way ANOVA. ****p ≤ 0.0001. **b** Biochemical analysis by western blot of the effect on PI3K and MAPK signaling in cells overexpressing SHP2. Significant differences (*p* ≤ 0.05) in p-SHP2, SHP2 and p-ERK in comparison to the untreated condition in parental cells are marked with an *. BYL719: 5 μM. **c** Long-term treatment with BYL719 of MCF7, T47D and MDA-MB-453 cells (for 38, 50 and 50 days, respectively) results in outgrowth of resistant clones. BYL719/SHP099 combination treatment prevents outgrowth of BYL719 resistant clones. Vehicle (UT) and SHP099-only treated cells serve as control. BYL719: 5 μM (MCF7), 2 μM (T47D) and 1 μM (MDA-MB-453). SHP099: 5 μM (MCF7 and MDA-MB-453), 10 μM (T47D). **d** Biochemical analysis of SHP2, PI3K and MAPK signaling of ten BYL719 resistant T47D clones. Parental cells were treated with BYL719 for 24 h. BYL719: 2 μM. Significant differences (*p* ≤ 0.05) in signaling in comparison to the untreated condition in parental cells are marked with an *. **e** Colony formation (top) and quantification of three colony formation experiments (bottom) of two BYL719-resistant T47D clones that are co-treated with BYL719 and SHP099. Significance between indicated conditions was calculated by one-way ANOVA. ****p ≤ 0.0001. BYL719: 2 μM. SHP099: 10 μM. **f** Biochemical effects of BYL719 (2 μM) and SHP099 (10 μM) combination treatment on PI3K and MAPK signaling in BYL719-resistant T47D clone #1. Significant differences (*p* ≤ 0.05) in signaling in comparison to the untreated condition in parental cells are marked with an *

To investigate whether SHP2 overexpression confers resistance to PI3K inhibition, we transfected MCF7 cells with a control vector, pCMV-EGFP, or a SHP2 wild type expression vector, pCMV-SHP2-WT. Levels of SHP2 expression increased markedly in stably transfected cells (Additional file 1: Fig. S2A). This overexpression of SHP2 led to increased colony formation, i.e., it did induce resistance to BYL719 (Fig. 2A, Additional file 1: Fig. S2B). ERK phosphorylation indicated markedly higher MAPK pathway activity in SHP2-overexpressing cells than MCF7 parental or pCMV-EGFP-transfected cells, and this coincided with a higher phosphorylation of SHP2 (Fig. 2B). We did not detect an effect on AKT signaling, indicating that resistance to BYL719 was due to high MAPK signaling.

Acquired drug resistance of cancer cells after continued exposure to medication is common in patients [50]. Therefore, we evaluated the role of SHP2 in acquired resistance to PI3K inhibition after prolonged exposure to BYL719. We seeded MCF7, T47D and MDA-MB-453 cells at low density, treated them with BYL719, SHP099 or a combination thereof, and allowed resistant colonies to form over time. Vehicle- and SHP099-treated cells quickly grew to confluence, while BYL719-treated cells formed numerous sizable colonies. In contrast, BYL719/SHP099-treated cells only grew as few small colonies, even after 38 or 50 days (Fig. 2C, Additional file 1: Fig. S2C). This result demonstrates that SHP2 is involved in the acquisition of resistance to BYL719.

Next, we closely studied the role of SHP2 in the development of acquired resistance to BYL719. We selected individual T47D and MDA-MB-453 cell clones that exhibited robust resistance to BYL719 after long-term treatment and confirmed their resistance to BYL719, compared to BYL719-sensitive parental cells (Additional file 1: Fig. S2D). To examine if SHP2 was involved in the BYL719-resistant phenotype, we subjected cell lysates of each clone to western blotting and measured phosphorylation of SHP2, ERK, AKT, S6 ribosomal protein and 4E-BP1. Importantly, SHP2 was clearly activated in about half of the BYL719-resistant clones (Fig. 2D, Additional file 1: Fig. S2E). In addition, most clones exhibited re-activation of ERK, AKT, S6 ribosomal protein or 4E-BP1. We probed the T47D clones for the phosphorylation of HER2 and HER3, two RTKs that are frequently activated upon BYL719 resistance [34, 51]. We observed that HER2 and HER3 were phosphorylated in several clones, particularly in conjunction with high SHP2 phosphorylation, suggesting a contribution to the resistant phenotype (Additional file 1: Fig. S2F). We selected two T47D clones with high levels of SHP2 phosphorylation to test the BYL719/SHP099 combination, which indeed reversed the BYL719 resistant phenotype (Fig. 2E). In BYL719-resistant T47D cells, co-treatment with BYL719 and SHP099 led to lower levels of phosphorylation of ERK, S6 ribosomal protein and 4E-BP1 compared to BYL719 monotreatment (Fig. 2F). These results confirm that SHP2 is a frequent and important mediator of acquired resistance to PI3K inhibition. Moreover, SHP2 is activated in cells that acquire resistance after prolonged exposure to PI3K inhibition.

### Targeting SHP2 effectively combats intrinsic resistance to PI3K inhibitors in TNBC cells

**Fig. 3:**
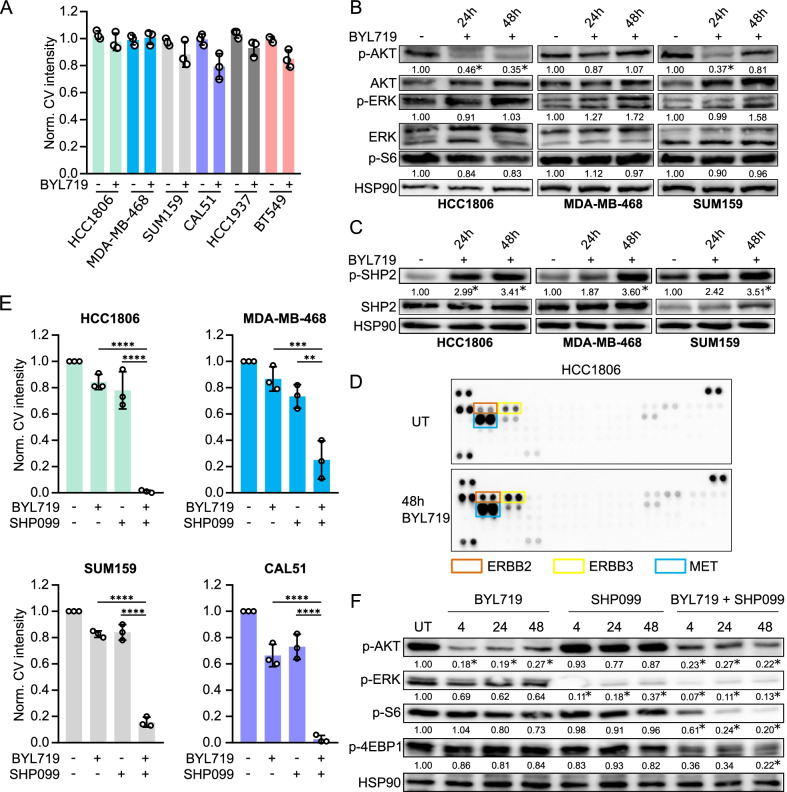
Targeting SHP2 effectively combats intrinsic resistance to PI3K inhibitor in TNBC cells. **a** A panel of PI3K inhibitor-resistant TNBC cell lines treated with BYL719. Quantification of crystal violet staining intensity of three independent colony formation experiments is shown. BYL719: 5 μM (HCC1806, MDA-MB-468, HCC1937, BT549), 2 μM (CAL-51, SUM159). **b** Western blot showing the biochemical effects on PI3K and MAPK signaling upon BYL719 treatment in HCC1806 (5 μM), MDA-MB-468 (5 μM) and SUM159 (2 μM) cells. Significant differences (*p* ≤ 0.05) in signaling in comparison to the untreated condition are marked with an *. **c** BYL719 treatment induces phosphorylation of SHP2 on residue Y542 in HCC1806 (5 μM BYL719), MDA-MB-468 (5 μM) and SUM159 (2 μM) cells. Significant differences (*p* ≤ 0.05) in induction of SHP2 phosphorylation in comparison to the untreated condition are marked with an *. **d** RTK arraying of HCC1806 cells treated with 5 μM BYL719 for 48 h reveals activation of several RTKs. **e** BYL719/SHP099 combination treatment in HCC1806, MDA-MB-468, SUM159 and CAL51 cells prevents resistance to BYL719. Quantifications of crystal violet staining intensity of three independent colony formation experiments. Significance between indicated conditions was calculated by one-way ANOVA. **p ≤ 0.01, ***p ≤ 0.001, ****p ≤ 0.0001. BYL719: 5 μM (HCC1806, MDA-MB-468), 2 μM (SUM159, CAL51). SHP099: 10 μM (HCC1806), 15 μM (SUM159), 20 μM (MDA-MB-468, CAL51). **f** PI3K and MAPK signaling is abrogated in HCC1806 cells that are treated with the combination of BYL719 and SHP099. BYL719: 5 μM. SHP099: 10 μM. Significant differences (*p* ≤ 0.05) in signaling in comparison to the untreated condition are marked with an *

Triple-negative breast cancers do not bear the classical hormonal targets and thus represent a difficult subgroup of breast cancer for rational therapies [52]. However, PI3K signaling is frequently aberrantly activated [2], but TNBC cells are often intrinsically resistant to PI3K inhibition. Therefore, we investigated whether SHP2 is a potential molecular target in TNBC. To validate our findings across multiple cell models, we collected a panel of six TNBC cell lines, each harboring mutations affecting PI3K signaling (Additional file 1: Table S1). These lines exhibited intrinsic resistance to BYL719 (Fig. 3A, Additional file 1: Fig. S3A), in contrast to the luminal breast cancer cell lines described above, which developed resistance as a consequence of exogenous growth factor treatments.

Biochemical analysis by western blotting revealed that BYL719 treatment in the TNBC cells did not sustainably suppress the phosphorylation of AKT and S6 ribosomal protein over time. In addition, ERK phosphorylation was readily detectable, indicating active MAPK pathway signaling (Fig. 3B, Additional file 1: Fig. S3B). Moreover, treatment with BYL719 induced SHP2 phosphorylation (Fig. 3C, Additional file 1: Fig. S3C). We used RTK arrays to examine RTK activity in HCC1806, MDA-MB-468 and SUM159 cells. We found that treatment with BYL719 led to upregulation of phosphorylation of several RTKs, including ERBB2, ERBB3 and MET in HCC1806, PDGFRα, VEGFR2 and INSR in MDA-MB-468 and IGF1R and INSR in SUM159 cells (Fig. 3D and Additional file 1: Fig. S3D, E). We used western blotting to confirm this effect for a number of RTKs (Additional file 1: Fig. S3F).

These results suggested involvement of RTK signaling and SHP2 in the BYL719-resistant phenotype and prompted us to test co-treatments of BYL719 and SHP099 in the cell line panel. Strikingly, we found that this combination was highly effective in stalling proliferation (Fig. 3E, Additional file 1: Fig. S3G). The same result was obtained when SHP099 was combined with an alternative PI3K inhibitor, GDC-0941, in HCC1806 and MDA-MB-468 cells (Additional file 1: Fig. S3H). These findings were corroborated by western blotting. PI3K and MAPK pathway activity were significantly lower in the BYL719/SHP099 combination treatment than for BYL719 monotreatment, as measured by the phosphorylation of AKT, S6 ribosomal protein, 4E-BP1 and ERK (Fig. 3F, Additional file 1: Fig. S3I).

**Fig. 4:**
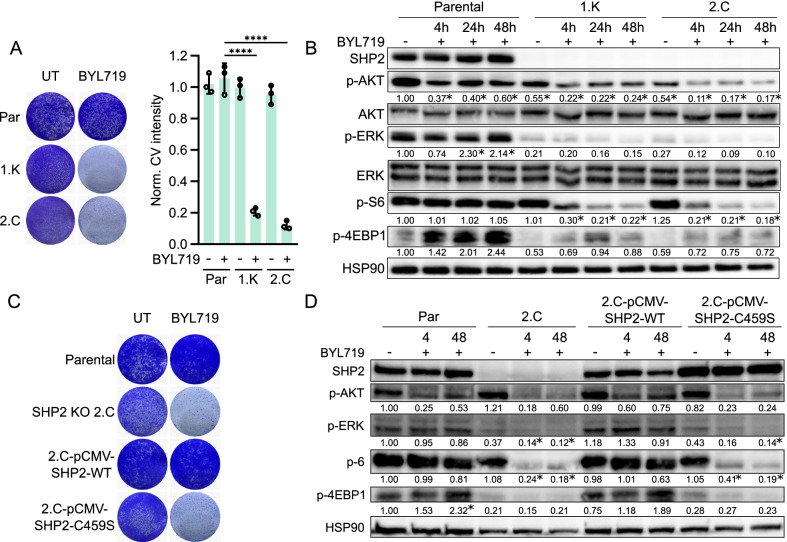
The phosphatase SHP2 is indispensable for mediating BYL719 resistance in TNBC cells. **a** Left: Colony formation with HCC1806 parental cells and two independent SHP2 knockout clones treated with 5 μM BYL719. Right: crystal violet intensity quantification of three experiments. Significance between indicated conditions was calculated by one-way ANOVA. ****p ≤ 0.0001. **b** Western blot analysis showing the biochemical effects on PI3K and MAPK signaling in HCC1806 parental versus SHP2 knockout cells after 5 μM BYL719 treatment. Significant differences (*p* ≤ 0.05) in signaling in comparison to the untreated condition in parental cells are marked with an *. **c** Colony formation with HCC1806 SHP2 knockout clone 2.C, reconstituted with either wild type SHP2 (pCMV-SHP2-WT) or a phosphatase-dead mutant (pCMV-SHP2-C459S), treated with 5 μM BYL719. HCC1806 parental and 2.C cells serve as controls. **d** Biochemical effects on PI3K and MAPK signaling pathways in HCC1806 parental, SHP2 KO 2.C and in wild type SHP2 and phosphatase-dead SHP2 reconstituted cells after treatment with 5 μM BYL719. Significant differences (*p* ≤ 0.05) in signaling in comparison to the untreated condition in parental cells are marked with an *

To ascertain that SHP2 is involved in the resistant phenotype, we used CRISPR-Cas9 technology to knock out *PTNP11* in HCC1806 and MDA-MB-468. We selected two SHP2 knockout clones for each cell line (Additional file 1: Fig. S4B). These clones were more sensitive to PI3K inhibition by BYL719 than parental HCC1806 and MDA-MB-468 cells (Fig. 4A, Additional file 1: Fig. S4A). We also evaluated the biochemical effects of knocking out SHP2 and simultaneously inhibiting PI3K in HCC1806 cells. Both PI3K and MAPK signaling were inhibited, as measured by the phosphorylation of AKT, S6 ribosomal protein, 4E-BP1 and ERK (Fig. 4B). This effect was similar to pharmacological inhibition of SHP2 using SHP099.

The phosphatase domain of SHP2 is important for the full function of SHP2, particularly for the activation of the signaling pathways it governs [53]. To test the importance of the phosphatase domain for resistance to PI3K inhibition, we reconstituted HCC1806 SHP2 knockout cells with either wild type SHP2 or a phosphatase-impaired mutant, SHP2-C459S [53]. Levels of SHP2 protein in the SHP2-reconstituted clones were comparable to those of the parental HCC1806 cells (Additional file 1: Fig. S4C). Upon treatment with BYL719, wild type SHP2-reconstituted cells exhibited a PI3K inhibitor-resistant phenotype similar to that of parental HCC1806 cells. In contrast, mutant SHP2-reconstituted cells were sensitive to PI3K inhibition, as was the original SHP2 knockout clone (Fig. 4C, Additional file 1: Fig. S4D). Western blotting confirmed sustained PI3K and MAPK signaling in wild type SHP2-reconstituted cells after BYL719 treatment, as seen in parental HCC1806 cells. Conversely, in mutant SHP2-reconstituted cells, BYL719 treatment robustly suppressed PI3K and MAPK signaling, as seen by the low levels of phosphorylation of AKT, S6 ribosomal protein, 4E-BP1 and ERK (Fig. 4D). In summary, these results show that TNBC cells, which are intrinsically resistant to PI3K inhibition, activate RTK signaling upon PI3K inhibitor treatment, and this re-activates PI3K and MAPK signaling. Moreover, combination treatment with SHP2 and PI3K inhibitors counteracts this process and blocks the proliferation of TNBC cells. Furthermore, an intact SHP2 phosphatase moiety is critical for conferring resistance to PI3K inhibition.

### Dual inhibition of PI3K and SHP2 blocks organoid formation in a patient-derived breast cancer model and tumor growth in a xenograft model

**Fig. 5:**
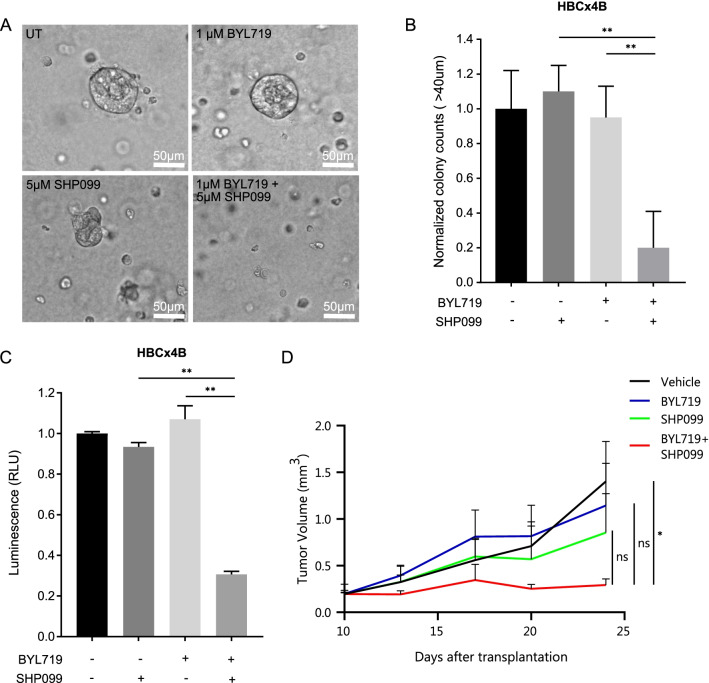
Dual PI3K/SHP2 inhibition blocks 3D organoid formation in two patient-derived breast cancer models and tumor growth in a xenograft model. **a** Representative images of organoids grown from patient-derived *PIK3CA* mutant HBCx4B TNBC cells after 10 days of treatment with BYL719, SHP099 or the combination thereof with the indicated concentrations. Magnification: 400×. **b** Normalized quantification of HBCx4B organoid formation in (**a**). Organoid diameter of 40 μm was taken as cut-off. Inhibition of organoid formation in the BYL719/SHP099 combination treatment is significant (*p* ≤ 0.01), calculated by one-way ANOVA. Experiment was performed on two biological replicates. **c** Normalized luminescence after CellTiter-Glo cell viability assay on the HBCx4B organoids of (**a**). Organoids treated with BYL719/SHP099 have significantly lower luminescence (*p* ≤ 0.01) compared to the monotreatments, calculated by one-way ANOVA. RLU: Relative Light Unit. Experiment was performed on three biological replicates. **d** Average tumor volume over time in nude mice injected with 2 × 10^6^ HCC1806 cells, treated with either vehicle, 15 mg/kg BYL719, 75 mg/kg SHP099 or the combination of 15 mg/kg BYL719 + 75 mg/kg SHP099. Three mice per group were treated daily starting at day 10 after injection. Inhibition of tumor growth in the BYL719/SHP099 combination treatment is significant (*p* ≤ 0.05), calculated by one-way ANOVA

Organoids represent more accurate models of human pathologies than cancer cells in culture, because they faithfully preserve the biological characteristics of tumors [54]. Organoids are 3D clusters derived from single stem cells that can be used as preclinical models to develop efficient new treatment regimens. Here, we made use of two human organoid cancer models, HBCx4B and HBCx60 [43], derived from two patient-derived xenograft triple-negative breast tumors that harbor *PIK3CA* mutations, E545K and H1047R, respectively. Single HBCx4B and HBCx60 cells were seeded in Matrigel, where they readily formed 3D organoids. We treated the growing organoids with BYL719, SHP099, and the combination thereof. Monotreatment with both compounds had little effect on the formation of organoids. However, the dual treatment significantly blocked the formation of organoids (Fig. 5A, B, Additional file 1: Fig. S5A, B). A cell viability test with CellTiter-Glo revealed that BYL719/SHP099-treated organoids were largely devoid of living cells in comparison to the other treatments, indicating a cytotoxic effect of the dual treatment (Fig. 5C, Additional file 1: Fig. S5C).

To validate the efficacy of combined PI3K/SHP2 inhibition in vivo, we made use of an HCC1806 xenograft model in mice. We injected 2 × 10^6^ cells into the cleared mammary fat pads of female nude mice. Tumors were allowed to grow for 10 days, reaching sizes of ~ 200 mm^3^, and then treatment was started. Tumors grew readily in vehicle-, BYL719- and SHP099-treated mice, but tumor growth in mice treated with BYL719 and SHP099 simultaneously was virtually absent (Fig. 5D). Mice treated with the BYL719/SHP099 combination exhibited good overall health, and the only negative result of the treatment seemed to be a small and only temporary effect on body weight, indicating low toxicity (Additional file 1: Fig. S5D). Altogether, these data in two clinically relevant breast cancer models highlight the potential of treatments that combine PI3K and SHP2 inhibitors.

## Discussion

PI3K inhibitors are in clinical development as agents directed at tumors with activated PI3K/AKT signaling. However, their usefulness is limited because cancer cells develop resistance to these drugs [36]. This explains why luminal breast tumors often exhibit a positive initial response to such therapies, but all too frequently persist. Our work yields essential insights into the mechanisms underlying PI3K inhibitor resistance, which often involves activation of receptor tyrosine kinase signaling, and identifies SHP2 as a crucial factor in this process in breast cancer cells. Working with luminal and triple-negative breast cancer cell line models, in organoids and in tumor-bearing mice, we show that co-inhibition of PI3K and SHP2 potently counteracts both acquired and intrinsic resistance to PI3K inhibitors. Our data suggest that this combined strategy might lead to the development of effective treatments for luminal tumors that have become resistant to PI3K inhibitors. Triple-negative breast cancers have been particularly difficult to treat, since they lack obvious molecular vulnerabilities that can be targeted [4]. They also generally exhibit an intrinsic resistance to PI3K inhibitors. Using several models of TNBC, we demonstrate that dual PI3K/SHP2 inhibition has a potent impact on blocking the proliferation of these tumor cells. This emphasizes the potential for novel therapies in triple-negative breast cancer patients based on combinations of PI3K and SHP2 inhibitors.

Our data move findings in other types of tumors into the arena of breast cancer. Sun et al. [55] have shown that simultaneously inhibiting SHP2 and PI3K is an effective approach in treating high-grade serous ovarian cancers that overexpress the GAB2 protein. This suggests that a strategy based on combined SHP2/PI3K inhibition might also be effective in other tumor types, but so far the foundation for extending it to breast cancer had yet to be established. Our work now puts this approach on a firm footing, and it would be of great interest to explore the combination treatment in further types of tumors that frequently exhibit aberrations in PI3K signaling, including colon, hepatocellular and gastric cancers [56].

Numerous RTKs have the potential to mediate drug resistance, and simultaneous activation of multiple RTKs may act as mechanisms of drug resistance [48, 49]. As a switch that controls signal transduction downstream of many RTKs and cytokine receptors, SHP2 appears to be an excellent target when the goal is to overcome diverse drug resistance mechanisms mediated by RTKs. At the same time, SHP2 has important functions in signal transduction in healthy cells, which means that using SHP2 inhibitors in patients could lead to toxicity. In animals, toxicity seems to be limited, as seen in the in vivo experiment presented here, as well as previous experiments by us and others [25, 26, 29]. Whether this is also the case in humans will need to be confirmed in clinical trials with SHP2 inhibitors. We are currently aware of two clinical trials that are testing novel SHP2 inhibitors in phase I studies (ClinicalTrials.gov Identifier: NCT03114319 and NCT04000529). If these studies find the drug to be tolerable, it would be highly interesting to extend the approach to clinical studies that combine SHP2 inhibitors with PI3K, MEK, BRAF or ALK inhibitors in breast cancer and further types of tumors which exhibit aberrations in the associated pathways. On the basis of our pre-clinical work and that of others [24–26, 31], we expect that combination strategies with SHP2 inhibitors are likely to be very powerful in patients.

The re-activation of pathways that have been inhibited and the activation of parallel signaling modes are recurring themes in studies of the mechanisms that underlie drug resistance to monotherapies [11]. Combination treatments can potentially overcome this problem by potent inhibition of multiple pathways. We show here that the parallel activation of MAPK signaling is mediated by RTKs and is instrumental in conveying PI3K inhibitor resistance. We demonstrate that co-inhibiting SHP2 effectively abrogates RTK-mediated MAPK signaling and restores the effects of PI3K inhibition. This supports previous proposals that combining PI3K and MEK inhibitors might offer a route to treating breast tumors [51, 57]. However, targeting SHP2 likely has important benefits compared to MEK, because it prevents the RTK-mediated re-activation of PI3K signaling and concurrently inhibits signaling pathways activated by RTKs, such as Src signaling, that may contribute to PI3K inhibitor resistance [40, 58].

SHP2 activates signal transduction downstream of RTKs via its phosphatase domain. SHP2 may enhance MAPK pathway activity by dephosphorylating negative regulators, such as Sprouty [59]. Moreover, SHP2 directly removes an inhibiting phosphorylation on RAS, boosting its activity [60]. Our data indicate that the phosphatase domain of SHP2 must be intact to mediate resistance to PI3K inhibitors. SHP2 knockout cells reconstituted with a phosphatase-dead SHP2 mutant are unable to (re-)activate AKT and MAPK signaling upon treatment with BYL719, which makes them sensitive to PI3K inhibition. This is in line with observations that a functional SHP2 phosphatase domain is required for RTK-mediated resistance to BRAF and MEK inhibitors [24, 25]. Combination treatments with potent catalytic SHP2 inhibitors may therefore be attractive alternatives to allosteric SHP2 inhibitors in fighting RTK-mediated resistance to kinase inhibitors.

## Conclusions

We show here that targeting SHP2 prevents RTK-mediated resistance to PI3K inhibitors in breast cancer. In luminal breast cancer, this approach overcomes acquired resistance to PI3K inhibition. In triple-negative breast cancer, this combination strategy could be a new, targeted therapeutic option to counteract intrinsic PI3K inhibitor resistance.

## Supplementary Information

**Additional file 1**. Figures S1-S5 and Table S1.

## Data Availability

For all data requests, please contact the corresponding author.

## Abbreviations

SHP2: Src Homology region 2 domain-containing Phosphatase 2
RTK: Receptor Tyrosine Kinase
PI3K: Phosphatidylinositol 3-Kinase
MAPK: Mitogen-Activated Protein Kinase
TNBC: Triple-negative Breast Cancer
EGF: Epidermal Growth Factor
NRG1: Neuregulin 1
FGF10: Fibroblast Growth Factor 10
ERBB2: Human Epidermal Growth Factor Receptor 2
ERBB3: Human Epidermal Growth Factor Receptor 3
MET: Hepatocyte Growth Factor Receptor
INSR: Insulin Receptor
IGF1R: Insulin-like Growth Factor 1 Receptor
PDX: Patient-Derived Xenograft
