# Supplementary material for: Targeting SHP2 phosphatase in breast cancer overcomes RTK-mediated resistance to PI3K inhibitors
Source: Breast Cancer Res. 2022 Apr 1;24:23. doi: 10.1186/s13058-022-01521-3 (PMC8974145; doi:10.1186/s13058-022-01521-3)
Supplement: Supplementary file 1 — Additional file 1. Figures S1-S5 and Table S1. [file 13058_2022_1521_MOESM1_ESM.pdf]

Figure S1

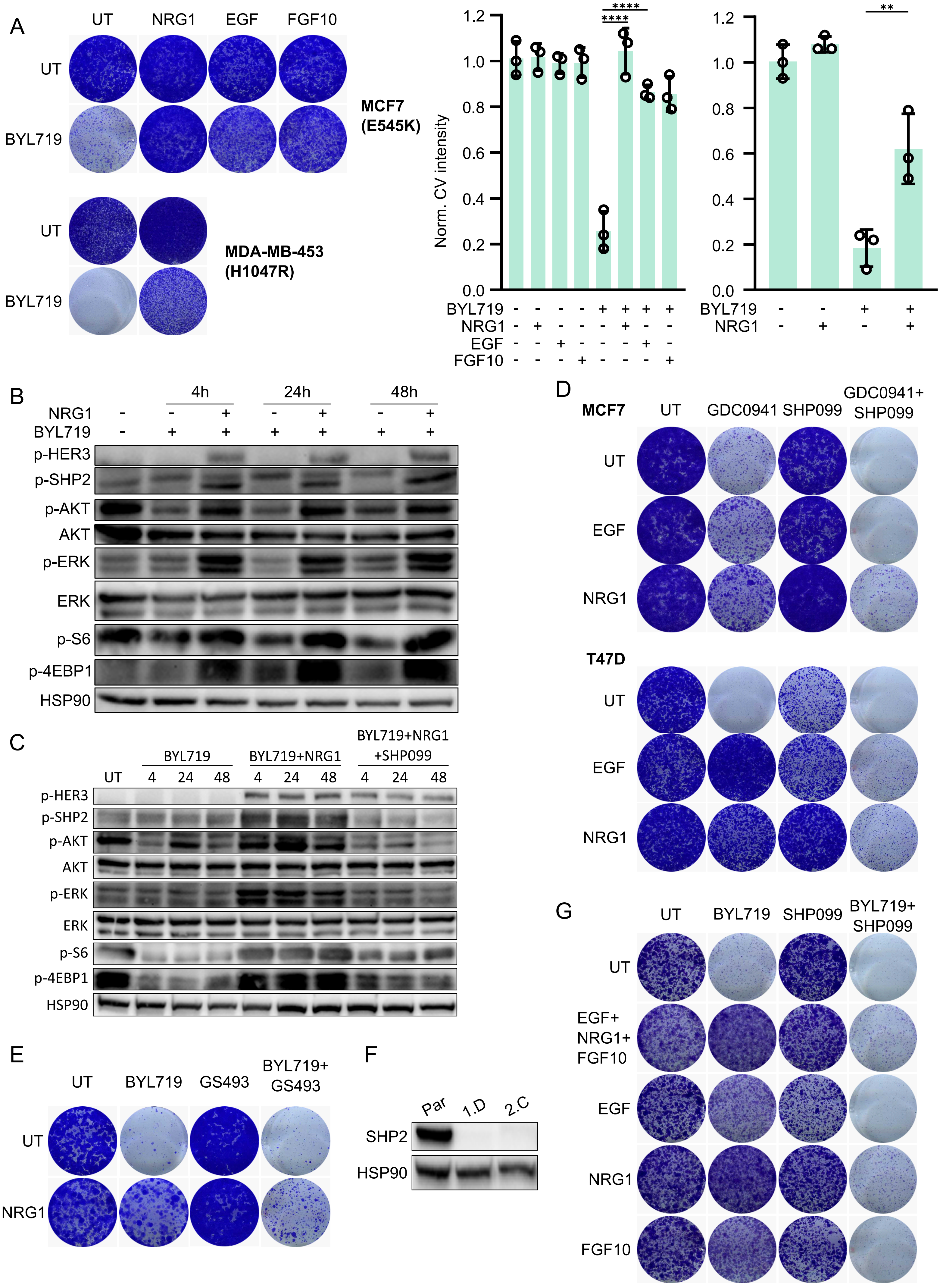
A
UT
NRG1
EGF
FGF10
UT
BYL719
MCF7 (E545K)
UT
BYL719
MDA-MB-453 (H1047R)
MCF7
MDA-MB-453
Norm. CV intensity
BYL719
NRG1
EGF
FGF10
B
4h
24h
48h
NRG1
BYL719
p-HER3
p-SHP2
p-AKT
AKT
p-ERK
ERK
p-S6
p-4EBP1
HSP90
C
BYL719
BYL719+NRG1
BYL719+NRG1 +SHP099
UT
4
24
48
D
MCF7
UT
GDC0941
SHP099
GDC0941+ SHP099
EGF
NRG1
T47D
E
GS493
BYL719+ GS493
F
Par
1.D
2.C
SHP2
HSP90
G
BYL719+ SHP099
EGF+ NRG1+ FGF10
FGF10

Supplementary Figure 1. Inhibition of SHP2 counteracts acquired resistance to PI3K inhibition in PI3K mutant breast cancer cells. A Multiple growth factors confer resistance to BYL719 in PI3K-mutant MCF7 and MDA-MB-453 breast cancer cells Left: colony formation. Right: quantification of crystal violet staining intensity of three independent colony formation experiments. Significance between indicated conditions was calculated by one-way ANOVA. **: $p \leq 0.01$, ****: $p \leq 0.0001$. BYL719: 5 µM (MCF7) and 1 µM (MDA-MB-453). NRG1, EGF and FGF10: 50 ng/ml. UT: vehicle-treated. **B** Western blot showing the biochemical effects on PI3K and MAPK signalling after BYL719 and NRG1 treatment in MCF7 cells. BYL719: 5 µM. NRG1: 50 ng/ml. **C** Western blot showing the biochemical effects on PI3K and MAPK signalling after BYL719, NRG1 and SHP099 treatment in MCF7 cells. BYL719: 5 µM. NRG1: 50 ng/ml. SHP099: 5 µM. **D** SHP099 reverses growth factor-induced resistance to PI3K inhibitor GDC0941 (pictilisib) in MCF7 and T47D cells. GDC0941: 0.5 µM. NRG1 and EGF: 50 ng/ml. SHP099: 10 µM. **E** The catalytic SHP2 inhibitor GS493 blocks growth-factor driven BYL719 resistance in MCF7 cells. BYL719: 5 µM. GS493: 20 µM. NRG1: 50 ng/ml. **F** Western blot confirming SHP2 knockout status in T47D clones 1.D and 2.C. **G** Co-treatment with SHP099 reverses resistance to BYL719 that is mediated by three simultaneously acting growth factors in MCF7 cells. BYL719: 5 µM. SHP099: 10 µM. NRG1, EGF and FGF10: 50 ng/ml.

Figure S2

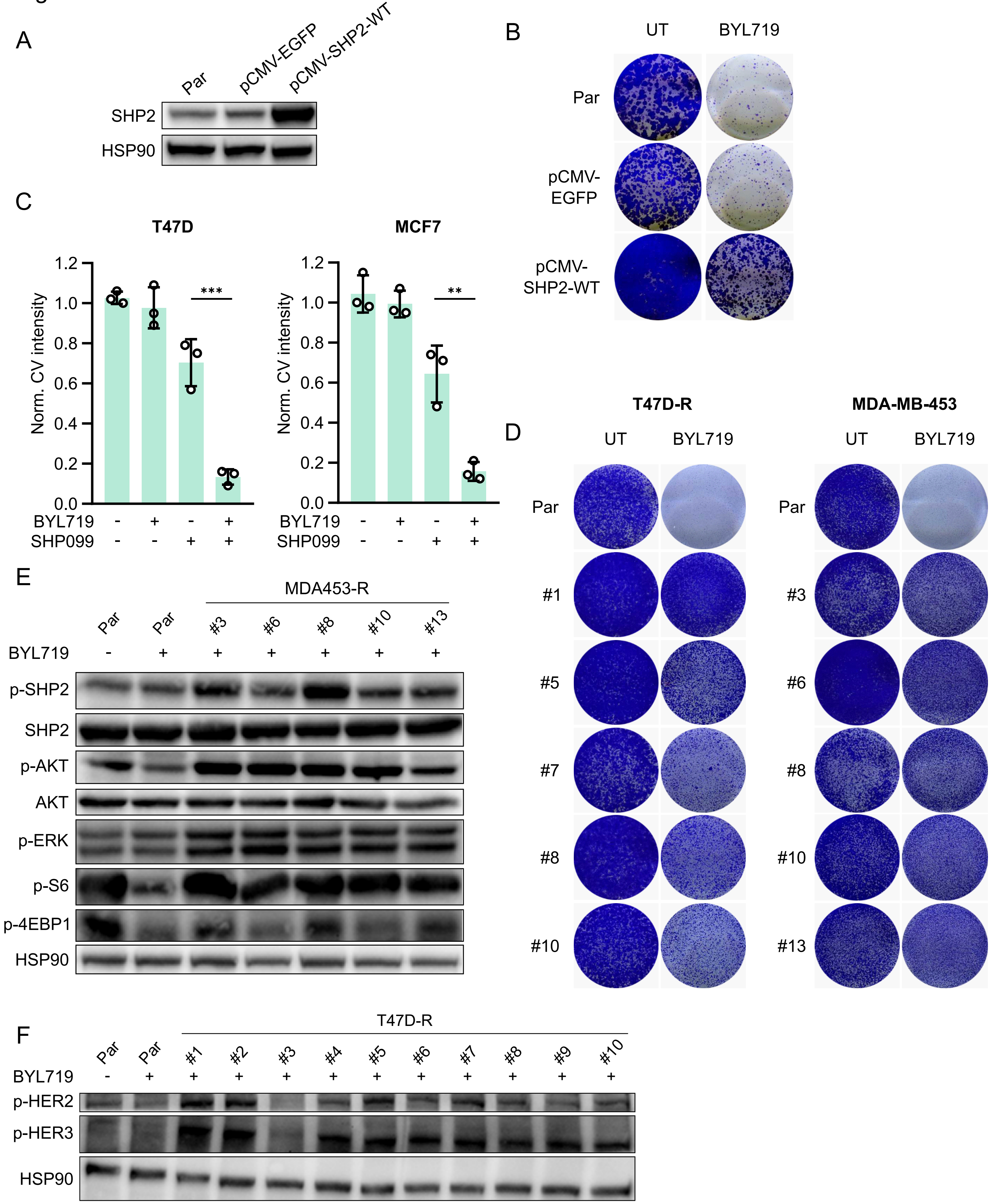

A
Par
pCMV-EGFP
pCMV-SHP2-WT
SHP2
HSP90
B
UT
BYL719
Par
pCMV-EGFP
pCMV-SHP2-WT
C
T47D
MCF7
Norm. CV intensity
1.2
1.0
0.8
0.6
0.4
0.2
0.0
***
**
BYL719 - + - +
SHP099 - - + +
D
T47D-R
MDA-MB-453
UT
BYL719
Par
#1
#5
#7
#8
#10
Par
#3
#6
#8
#10
#13
E
MDA453-R
Par
Par
#3
#6
#8
#10
#13
BYL719 - + + + + + +
p-SHP2
SHP2
p-AKT
AKT
p-ERK
p-S6
p-4EBP1
HSP90
F
T47D-R
Par
Par
#1
#2
#3
#4
#5
#6
#7
#8
#9
#10
BYL719 - + + + + + + + + + + +
p-HER2
p-HER3
HSP90

Supplementary Figure 2. SHP2 confers resistance to PI3K inhibition upon overexpression and long-term PI3K inhibitor exposure. **A** Western blot confirming overexpression of wild type SHP2 protein in MCF7-pCMV-SHP2-WT cells. **B** MCF7 cells overexpressing wild type SHP2 (pCMV-SHP2-WT) are resistant to BYL719 (5 μM) treatment. MCF7 parental and pCMV-GFP cells serve as control. **C** Quantification of crystal violet intensity of the MCF7 and T47D colony formations shown in Fig. 2C. **D** Colony formation assays of BYL719-treated T47D and MDA-MB-453 parental cells and five BYL719 resistant clones. BYL719: 2 μM (T47D) and 1 μM (MDA-MB-453). **E** Western blot analysis of five BYL719-resistant MDA-MB-453 clones evaluating SHP2 phosphorylation and ERK and PI3K pathway activation. Parental cells were treated with BYL719 for 24 hours. BYL719: 1 μM. **F** Phosphorylation of HER2 and HER3 in ten BYL719 resistant T47D clones. BYL719: 2 μM.

Figure S3

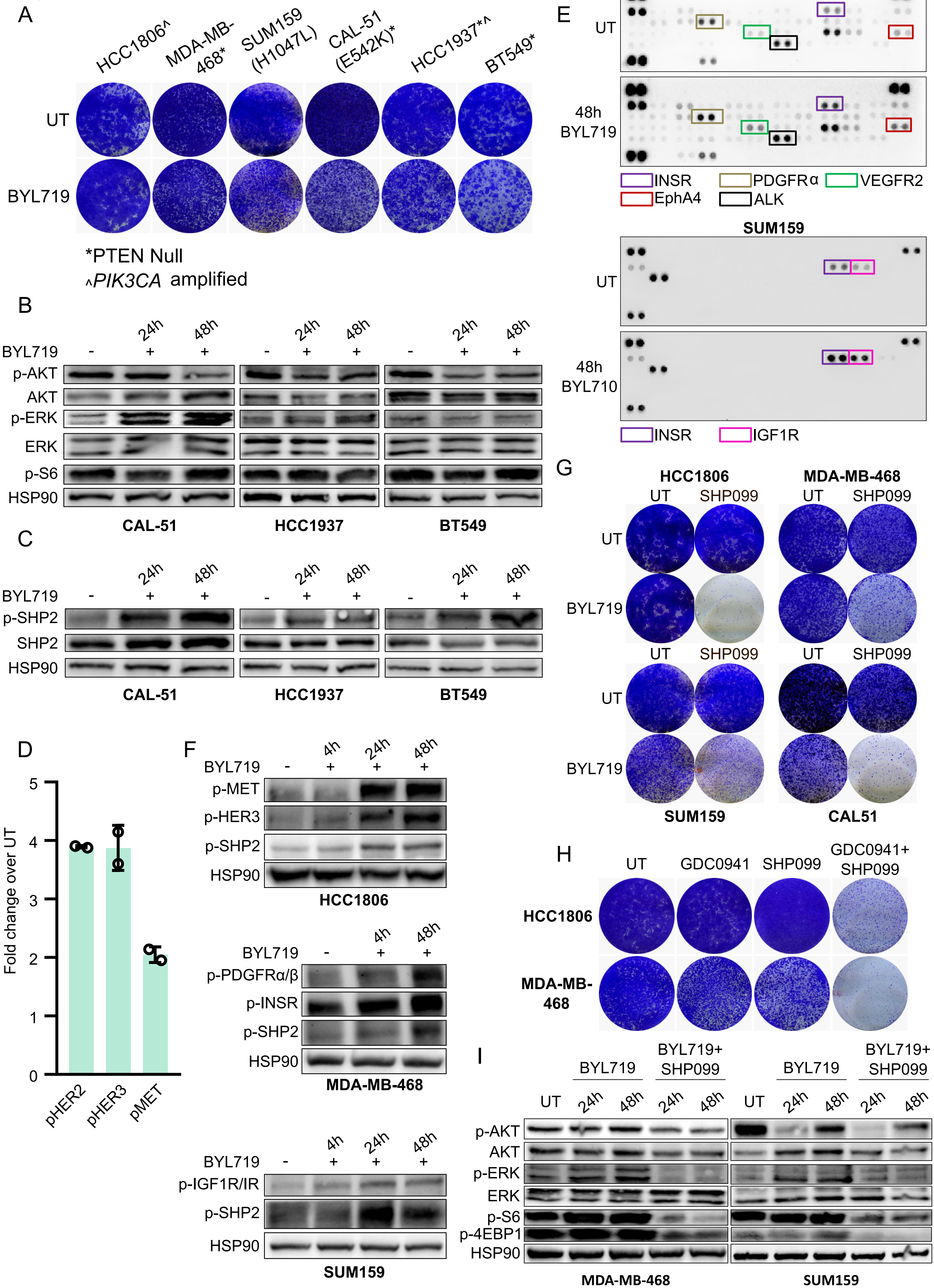
A
HCC1806^
MDA-MB-468*
SUM159 (H1047L)
CAL-51 (E542K)*
HCC1937*^
BT549*
UT
BYL719
*PTEN Null
^PIK3CA amplified
B
BYL719
24h
48h
p-AKT
AKT
p-ERK
ERK
p-S6
HSP90
CAL-51
HCC1937
BT549
C
p-SHP2
SHP2
D
Fold change over UT
pHER2
pHER3
pMET
E
MDA-MB-468
48h BYL719
INSR
PDGFRα
VEGFR2
EphA4
ALK
SUM159
48h BYL710
IGF1R
F
4h
p-MET
p-HER3
HCC1806
p-PDGFRα/β
p-INSR
MDA-MB-468
p-IGF1R/IR
SUM159
G
SHP099
CAL51
H
GDC0941
GDC0941+ SHP099
I
BYL719+ SHP099
p-4EBP1

Supplementary Figure 3. Targeting SHP2 effectively combats intrinsic resistance to PI3K inhibitor in TNBC cells. A Colony formations of untreated and BYL719 treated HCC1806 (5 µM), MDA-MB-468 (5 µM), SUM159 (2 µM), CAL-51 (2 µM), HCC1937 (5 µM) and BT549 (5 µM) cells. Relevant PI3K-related mutations are indicated. **B** Western blot showing the biochemical effects on PI3K and MAPK signaling upon BYL719 treatment in CAL-51 (2 µM), HCC1937 (5 µM) and BT549 (5 µM) cells. **C** Phosphorylation of SHP2 on residue Y542 is induced by BYL719 treatment in CAL-51 (2 µM), HCC1937 (5 µM) and BT549 (5 µM) cells. **D** Quantification of the upregulation of HER2, HER3 and MET phosphorylation after BYL719 treatment over vehicle treated HCC1806 cells of the RTK array of Fig. 3D. **E** Activation of RTK signalling, measured by RTK arrays, after 48 hours of BYL719 treatment in MDA-MB-468 (5 µM) und SUM159 (2 µM). **F** Validation by western blotting of the activation of several RTKs seen in the RTK arrays after treatment with BYL719 in HCC1806 (5 µM), MDA-MB-468 (5 µM) and SUM159 (2 µM) cells. **G** Representative colony formations from the crystal violet quantifications of Fig. 3E. **H** SHP099 counteracts intrinsic resistance to PI3K inhibitor GDC0941 (pictilisib) in HCC1806 and MDA-MB-468 cells. GDC0941: 1 µM. SHP099: 10 µM (HCC1806) and 15 µM (MDA-MB-468). **I** Inhibition of PI3K and MAPK signaling after BYL719/SHP099 combination treatment in MDA-MB-468 and SUM159 cells. BYL719: 5 µM (MDA-MB-468), 2 µM (SUM159). SHP099: 20 µM.

Figure S4

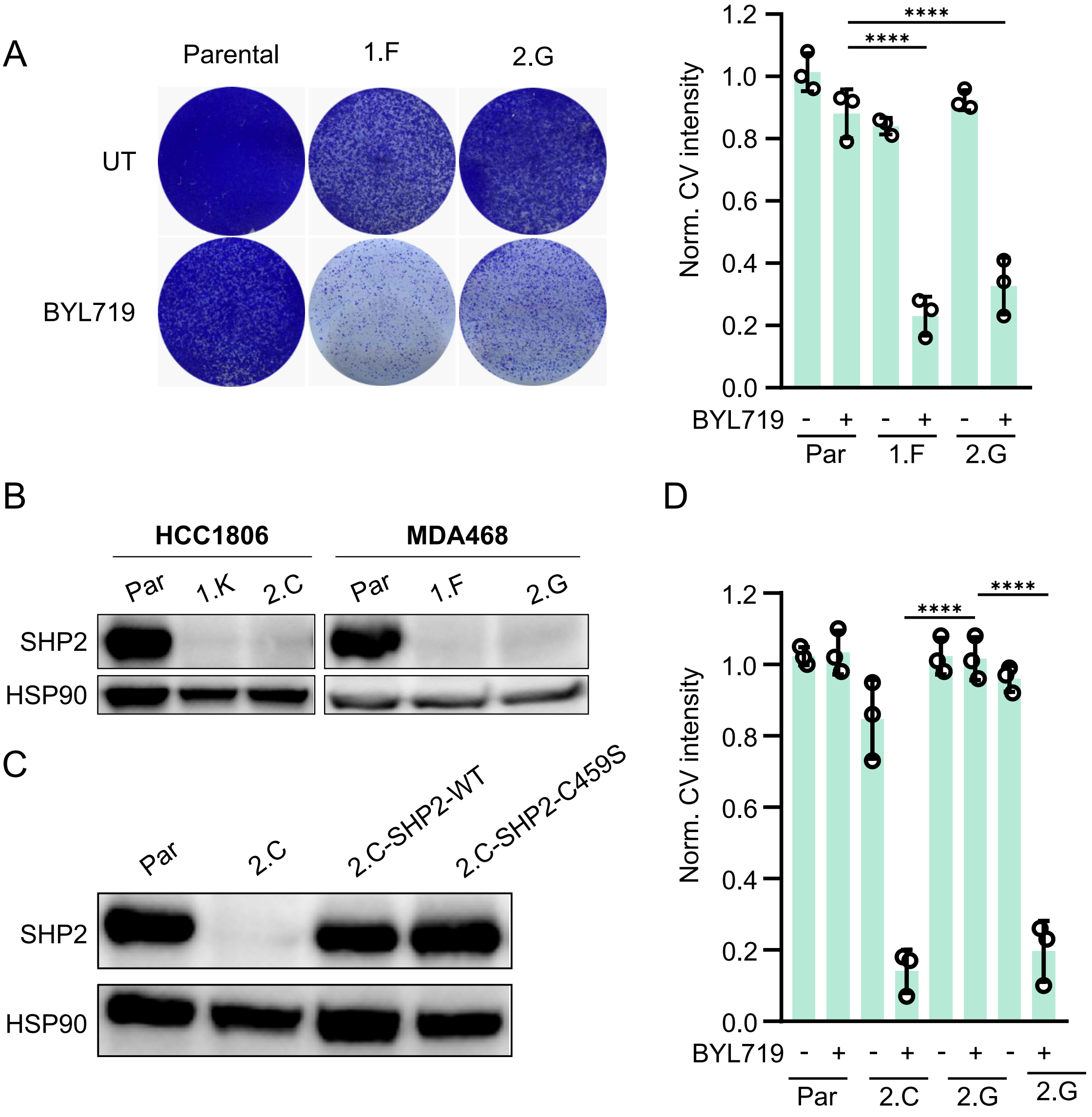
A
Parental
1.F
2.G
UT
BYL719
Norm. CV intensity
1.2
1.0
0.8
0.6
0.4
0.2
0.0
****
****
BYL719 - + - + - +
Par
1.F
2.G
B
HCC1806
MDA468
Par
1.K
2.C
Par
1.F
2.G
SHP2
HSP90
C
Par
2.C
2.C-SHP2-WT
2.C-SHP2-C459S
SHP2
HSP90
D
Norm. CV intensity
1.2
1.0
0.8
0.6
0.4
0.2
0.0
****
****
BYL719 - + - + - + - +
Par
2.C
2.G
2.G

Supplementary Figure 4. The phosphatase SHP2 is indispensable in mediating BYL719 resistance in TNBC cells. A Left: Colony formation with MDA-MB-468 parental cells and two independent SHP2 knockout clones treated with 5 μM BYL719. Right: crystal violet intensity quantification of three independent experiments is shown. **B** Western blot confirming that SHP2 protein expression is absent in HCC1806 and MDA-MB-468 SHP2 knockout clones. **C** SHP2 wild type and SHP2-C459S expression in reconstituted HCC1806-2.C cells, measured by western blotting. SHP2 expression in parental cells serves as comparison. **D** Quantification of three independent colony formation experiments as shown in Fig. 4C.

Figure S5

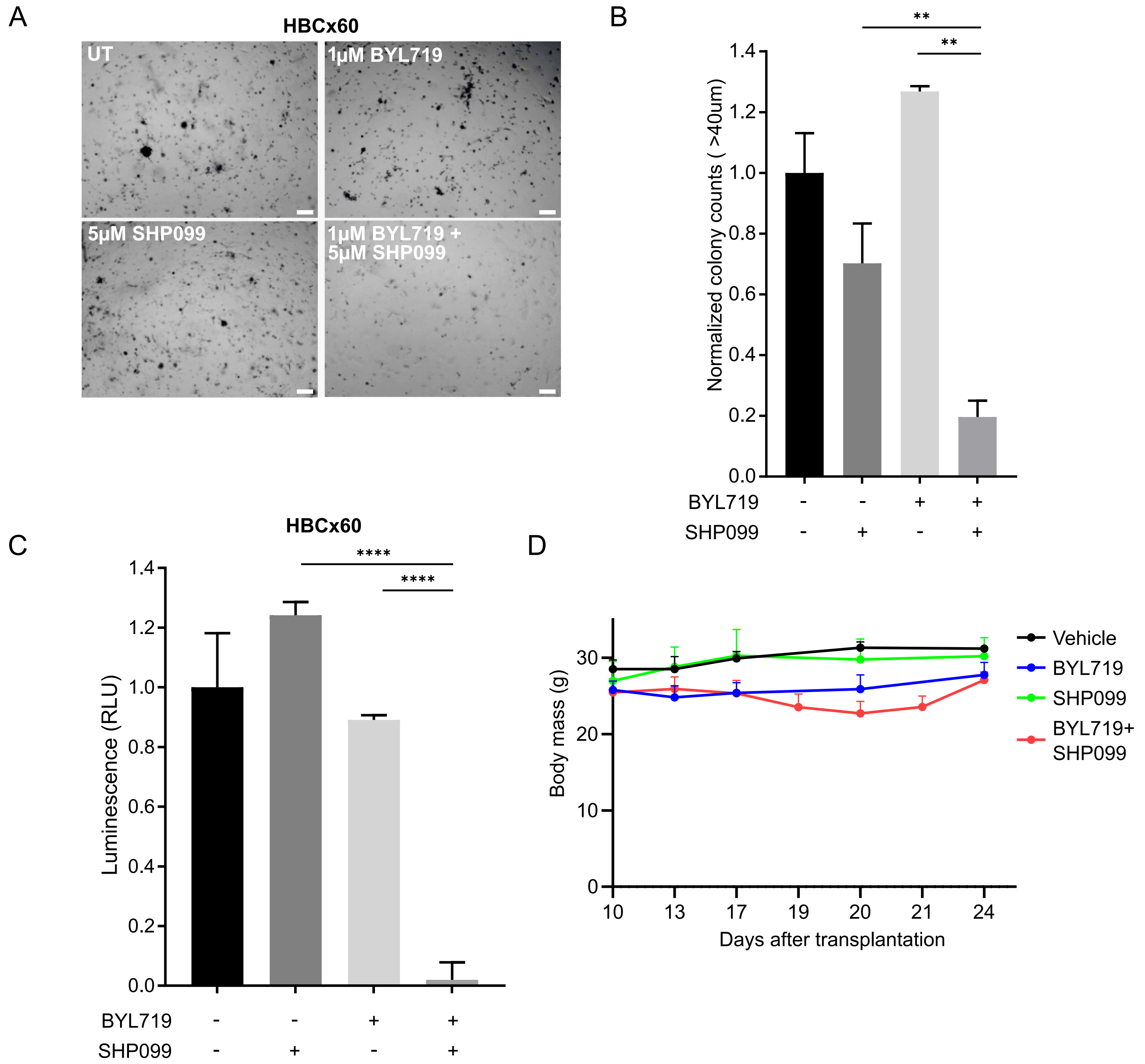

A
HBCx60
UT
1µM BYL719
5µM SHP099
1µM BYL719 +
5µM SHP099
B
HBCx60
Normalized colony counts (>40um)
1.4
1.2
1.0
0.8
0.6
0.4
0.2
0.0
**
**
BYL719 - - + +
SHP099 - + - +
C
HBCx60
Luminescence (RLU)
1.4
1.2
1.0
0.8
0.6
0.4
0.2
0.0
****
****
BYL719 - - + +
SHP099 - + - +
D
Body mass (g)
30
20
10
0
10 13 17 19 20 21 24
Days after transplantation
Vehicle
BYL719
SHP099
BYL719+
SHP099

Supplementary Figure 5. Dual PI3K/SHP2 inhibition blocks 3D organoid formation in two patient-derived breast cancer models and tumor growth in a xenograft model. **A** Representative images of organoids grown from patient-derived *PIK3CA* mutant HBCx60 TNBC cells after 10 days of treatment with BYL719, SHP099 or the combination thereof. Magnification: 100x. Scale bar: 100 µm. **B** Normalized quantification of HBCx60 organoid formation in Fig. S5A. Organoid diameter of 40 µm was taken as cut-off. Organoid formation in the BYL719/SHP099 combination treatment is significantly inhibited (**: $p \leq 0.01$), calculated by one-way ANOVA. Experiment was performed on two biological replicates. **C** Normalized luminescence after CellTiter-Glo cell viability assay on the HBCx60 organoids of Fig. S5A. Organoids treated with BYL719/SHP099 have significantly lower luminescence (****: $p \leq 0.0001$) compared to the monotreatments, calculated by one-way ANOVA. RLU: Relative Light Unit. Experiment was performed on three biological replicates. **D** Average body mass over time for all four treatment groups of HCC1806 xenografted mice of Fig. 5D, 3 mice per group.

Cell line	Subtype	ER	PR	HER2	PI3K	PTEN	Culture medium
T47D	Luminal	+	+	-	H1047R	WT	DMEM+10% FCS+5 μg/ml ins+1% PS
MCF7	Luminal	+	+	-	E545K	WT	DMEM+10% FCS+5 μg/ml ins+1% PS
MDA-MB-453	TNBC	-	-	+	H1047R	WT	DMEM+10% FCS+1% NEAA+1% PS
HCC1806	TNBC	-	-	-	Amplified	WT	RPMI+10% FCS+1% PS
MDA-MB-468	TNBC	-	-	-	WT	Null	DMEM+10% FCS+1% NEAA+1% PS
SUM159	TNBC	-	-	-	H1047L	WT	Ham/F12+10% FCS+1% Hydrocortisone+ 5 μg/ml ins+10 mM HEPES+1% PS
CAL-51	TNBC	-	-	-	E542K	Null	DMEM+10% FCS+1% NEAA+1% PS
HCC1937	TNBC	-	-	-	Amplified	Null	RPMI+10% FCS+1% PS
BT549	TNBC	-	-	-	WT	Null	DMEM+10% FCS+1% NEAA+1% PS

Supplementary Table 1. Relevant characteristics and mutations of used breast cancer cell lines [40, 41].
